# Characteristics and Outcomes of Adults Hospitalized With Childhood-Onset Complex Chronic Conditions

**DOI:** 10.1001/jamanetworkopen.2025.53610

**Published:** 2026-01-28

**Authors:** Sarah L. Malecki, Trong Shen, Anne Loffler, Therese A. Stukel, Claire de Oliveira, Surain B. Roberts, Anne S. Bassett, Kate E. Nelson, Fahad Razak, Amol A. Verma, Eyal Cohen

**Affiliations:** 1 Child Health Evaluative Sciences, SickKids Research Institute, Toronto, Ontario, Canada; 2 Division of General Internal Medicine, University of Toronto, Toronto, Ontario, Canada; 3 Institute of Health Policy, Management and Evaluation, Dalla Lana School of Public Health, University of Toronto, Toronto, Ontario, Canada; 4 Edwin S. H. Leong Centre for Healthy Children, University of Toronto, Toronto, Ontario, Canada; 5 Li Ka Shing Knowledge Institute, St Michael’s Hospital, Toronto, Ontario, Canada; 6 ICES, Toronto, Ontario, Canada; 7 Institute for Mental Health Research, Centre for Addiction and Mental Health, Toronto, Ontario, Canada; 8 Campbell Family Mental Health Research Institute, Centre for Addiction and Mental Health, Toronto, Ontario, Canada; 9 Clinical Genetics Research Program, Centre for Addiction and Mental Health, Toronto, Ontario, Canada; 10 The Dalglish Family 22q Clinic, University Health Network, Toronto, Ontario, Canada; 11 Department of Psychiatry, University of Toronto, Toronto, Ontario, Canada; 12 Toronto General Hospital Research Institute, University Health Network, Toronto, Ontario, Canada; 13 Division of Paediatric Medicine, The Hospital for Sick Children, Toronto, Ontario, Canada

## Abstract

**Importance:**

Individuals with childhood-onset complex chronic conditions (4Cs) are increasingly surviving to adulthood, but the impacts of these conditions on the adult health care system is largely unknown.

**Objective:**

To identify and characterize young adults with 4Cs in hospitals and compare their outcomes with other hospitalized young adults.

**Design, Setting, and Participants:**

This retrospective cohort study included young adults aged 18 to 39 years who were discharged from 29 hospitals in Ontario, Canada, from January 1 to December 31, 2018. Analyses were performed from November 2024 to April 2025.

**Exposure:**

Childhood-onset complex chronic conditions, identified using an adaptation of a widely used pediatric algorithm.

**Main Outcomes and Measures:**

Primary outcomes included hospital length of stay, in-hospital mortality, and intensive care unit (ICU) admissions. Secondary outcomes were total cost, number of medications, number of advanced imaging scans, and 30-day readmission rates. Regression weighted by propensity score–based overlap weights was used to assess the association of 4Cs with outcomes at the patient level.

**Results:**

Of 19 915 hospitalizations experienced by 15 072 patients (49.6% male; median age, 30 years; interquartile range, 24-35 years), 1329 (6.7%) were associated with 4Cs in 814 individuals (5.4%). The most prevalent pediatric conditions were hereditary anemias (212 of 814 individuals [26.0%]), cystic fibrosis (138 of 814 [17.0%]), and cerebral palsy (96 of 814 [11.8%]). Hospitalizations of patients with 4Cs accounted for 13 606 of 126 910 young adult hospital bed-days (10.7%). Patients hospitalized with 4Cs had lower Charlson Comorbidity Index scores (eg, ≥3: 7 of 757 [0.9%] vs 381 of 13 547 [2.8%]) and were more likely to have longer hospital stays (relative ratio [RR], 1.62; 95% CI, 1.48-1.77), higher total costs (RR, 1.65; 95% CI, 1.05-2.59), more prescribed medications (RR, 1.26; 95% CI, 1.19-1.34), fewer advanced imaging scans (RR, 0.85; 95% CI, 0.77-0.93), and higher 30-day readmission rates (RR, 1.59; 95% CI, 1.28-1.98) compared with those without 4Cs. No significant differences were observed among those with vs without 4Cs for ICU admissions (RR, 1.05; 95% CI, 0.91-1.20) or in-hospital mortality (RR, 1.43; 95% CI, 0.87-2.33).

**Conclusions and Relevance:**

In this cohort study, young adults with 4Cs had longer acute care medical hospitalizations, accounting for 10.7% of all young adult hospital bed-days, and more rehospitalizations than other young adults. The results suggest that this population should be prioritized for targeted measures to reduce inpatient stays and improve care. The Charlson Comorbidity Index may be of limited utility for capturing comorbidities in this population.

## Key Points

**Question:**

What are the characteristics and outcomes of hospitalizations of young adults with childhood-onset complex chronic conditions (4Cs)?

**Findings:**

In this cohort study of 19 915 hospitalizations among 15 072 young adults (aged 18-39 years), 4Cs were present in 6.7% of hospitalizations, 10.7% of hospital bed-days, and 5.4% of individuals. Hospitalizations of patients with 4Cs involved longer lengths of stay and greater costs and readmission rates compared with hospitalizations of patients without 4Cs.

**Meaning:**

The findings suggest that patients with 4Cs should be prioritized in adult hospitals for targeted measures to reduce inpatient stays and improve care.

## Teaser

This cohort study evaluates the characteristics and outcomes of hospitalizations of young adults aged 18 to 39 years with childhood-onset complex chronic conditions.

## Introduction

Childhood-onset complex chronic conditions (4Cs) are characteristic, persistent medical conditions that involve multiple organ systems and/or require specialized care.^1,2,3^ Individuals with 4Cs make up a disproportionate share of pediatric health care, particularly spending within inpatient settings.^4^ Disease-agnostic multidisciplinary complex care teams have emerged in many children’s hospitals to address unmet needs and care coordination.^5^ Individuals with 4Cs are increasingly surviving to adulthood due to advances in pediatric care.^6,7^ However, outcomes beyond age 20 years^1,8^ in resource-intensive acute care settings remain largely undescribed for this group as a whole.

Prior studies^9,10,11^ of adults with conditions originating in childhood did not adequately allow assessment of outcomes for individuals with 4Cs. A small number of complex conditions without a comparator have been examined.^9,10^ One study described higher resource use for adults with pediatric-onset disability,^11^ defined as any intellectual or physical functioning impairment or developmental process abnormality, including congenital malformations that are relatively common and would not be considered complex (eg, simple ventricular septal defects). That study also excluded important 4Cs that are not captured under the congenital malformations categories of international coding schemes (eg, sickle cell disease and cystic fibrosis) and was not population based, limiting its generalizability.^11^

Adult hospital outcome data for 4Cs is needed to estimate the impact in this emerging population. Health administrative data from adult hospitals in systems with universal health insurance coverage and where all children transfer to adult care at age 18 years can provide a comprehensive picture of this impact from a population health perspective. Our aim was to identify, characterize, and assess acute care hospitalizations of young adults with 4Cs in terms of total numbers and outcomes. Given the disproportionate impact on hospital spending for these conditions in childhood,^4^ our primary hypothesis was that young adults with 4Cs would have higher hospital resource use than young adults without 4Cs.

## Methods

### Design and Setting

This retrospective cohort study used data from GEMINI (formerly the General Medicine Inpatient Initiative) research network,^12,13^ which includes clinical and administrative data for all adult medical and intensive care unit (ICU) admissions at approximately 30 hospitals in Ontario, Canada. Data are extracted from hospital information systems,^14^ demonstrating 98% to 100% agreement compared with manual medical record review.^15^ We restricted our analysis to data available prior to the COVID-19 pandemic (January 1 to December 31, 2018; 29 hospitals) given the known disruption in regular health service use during the pandemic and associated challenges with interpreting hospitalization data. Data analysis was performed from November 2024 through April 2025. We further limited our analysis to hospitalizations for young adults aged 18 to 39 years^16^ to increase the specificity of our cohort definition given our focus on physical health conditions usually diagnosed in childhood but without a look-back period to identify pediatric onset or diagnosis. The Strengthening the Reporting of Observational Studies in Epidemiology (STROBE) reporting guideline was followed. The study was approved by the Unity Health and University of Toronto research ethics boards, with waiver of informed consent provided because of use of retrospective, deidentified data.

### Cohort and Comparator

We identified unique patients with a valid health card number to allow linking of individuals across hospital encounters. To identify 4Cs in each encounter, we adapted an algorithm commonly used to identify children with complex chronic conditions (CCCs).^1,3^ The algorithm included *International Statistical Classification of Diseases and Related Health Problems, Tenth Revision (ICD-10)* codes indicating the presence of a CCC (the eMethods in Supplement 1 gives more details). It was necessary to further adapt this algorithm for use in adult hospitalization data because many CCCs are not specific to childhood (eg, epilepsy) and chronic condition diagnoses recorded in hospital discharge data do not have a date of diagnosis. Linkable pediatric data were not available to determine age at onset.

To adapt the algorithm, 2 clinicians (a general internist [S.L.M.] and a pediatric hospitalist [E.C.]) first categorized each *ICD-10* code in the pediatric CCC algorithm^1,3^ into 3 categories: most likely pediatric (eg, cystic fibrosis), possibly pediatric (eg, inflammatory bowel disease), and likely not pediatric (eg, Parkinson disease). Disagreements were resolved by another one of us (K.E.N., a pediatrician with experience using CCC codes). The full list of *ICD-10* codes in the CCC algorithm and the pediatric categorization applied to adult data are further described in the eAppendix in Supplement 1 and the eTable in Supplement 2.

For the main analysis, the cohort with 4Cs was defined by 1 or more *ICD-10* codes in any discharge diagnosis field that indicated a CCC that, based on its description, most likely began in childhood. Patients with hospitalizations without any *ICD-10* code categorized as most likely pediatric were used as the comparator cohort. We opted to take a conservative approach in our primary analysis, using only most likely pediatric codes to define 4Cs. Accordingly, all possibly pediatric codes were included in the comparator group. A sensitivity analysis using a less conservative approach defined 4Cs as most likely pediatric or possibly pediatric *ICD-10* codes (eAppendix in Supplement 1).

### Characteristics

Characteristics collected at admission included age group (18-24, 25-29, and 30-39 years), sex, neighborhood income quintile (eMethods in Supplement 1), admission hospital and type (teaching vs community), illness severity as measured by the modified Laboratory-Based Acute Physiology Score (mLAPS) (eMethods in Supplement 1),^17,18^ and Charlson Comorbidity Index (CCI),^19,20^ a widely used morbidity measure that has been shown to predict hospital outcomes in older adults.^19^ For individuals with at least one 4C, we reported the most common 4C subcategories (per the original algorithm)^3^ and the number of 4C diagnoses. For hospitalizations with and without 4Cs, we reported the most common Charlson comorbidities^21^ and main discharge diagnoses as the individual code and grouped by Clinical Classifications Software Refined categories.^22^

### Outcomes

Our 3 coprimary outcomes included (1) total length of hospital stay, (2) in-hospital mortality, and (3) ICU admission during the hospitalization. Secondary outcomes included (1) total inpatient cost, as estimated by multiplying resource intensity weights (a measure of health care utilization) by cost of standard hospital stay (a unit cost)^23,24,25^; (2) number of inpatient medications; (3) number of advanced imaging scans (computed tomography or magnetic resonance imaging); and (4) 30-day readmissions to a medical or ICU service (the eMethods in Supplement 1 gives more details).

### Statistical Analysis

To describe the prevalence and characteristics of 4Cs in hospital data, and to estimate the association of 4Cs with hospital outcomes, we first described cohort characteristics at the level of the encounter (proportion of hospitalizations, number of 4Cs, and proportion of hospital bed-days). To eliminate clustering effects of repeated admissions and biasing results toward a small number of individuals with frequent hospitalizations, we selected the first (index) hospitalization to an adult hospital within the study time frame for each individual as the unit of analysis.

Some hospitals did not have complete laboratory, pharmacy, and cost data.^13^ For periods with laboratory data coverage, we assumed that patients admitted through the emergency department without laboratory tests did not have any indications for testing and, thus, had normal test results. Similarly, for periods with pharmacy data coverage, we assumed that patients without pharmacy orders did not receive any medication.

We reported standardized mean differences (SMDs) in characteristics between groups, with differences greater than or equal to 0.1 indicating important imbalance between groups.^26^ Propensity score–based overlap weights^27,28,29^ were then used to balance baseline characteristics (ie, characteristics before or at the time of exposure) between index hospitalizations with and without pediatric complexity. Although 4Cs were defined at hospital admission, this construct is not a point exposure like an intervention but is rather representative of chronic conditions that started in childhood, likely at birth in many cases (eg, cystic fibrosis, congenital syndromes). We thus estimated the propensity for pediatric complexity using multivariable random effects logistic regression,^29^ adjusting only for age, sex, and income quintile as covariates given their likely long-term presence. Hospital of admission was included as a random effect in the propensity score model to improve balance in the proportion of patients admitted to each hospital given that processes of care are expected to be more similar within hospitals.^29^ The propensity score model was used to generate overlap weights for each patient in the cohort, and each patient was weighted according to their overlap weight (ie, probability of being assigned to the opposite exposure group based on the propensity score).^28^

Univariate regression models were run to assess the association of 4Cs with outcomes. Length of stay was highly skewed, so linear regression was performed on the weighted log-transformed value. Similar to prior studies,^30,31^ we modeled total cost using a generalized linear regression model with gamma distribution. For the other outcomes, which were binary or counts, Poisson regression models were used after confirming no overdispersion. All models were weighted by overlap weights and included a robust variance estimator. Regression coefficients were exponentiated to derive the relative outcome ratio and 95% CI of the effect estimate. For all models, we found no issues with influential observations (Cook distance <2).^32^ Given the wide age range included in this study, we performed a sensitivity analysis to test the robustness of our results after restricting to a narrower age range (18-24 years).

All analyses were performed in R, version 4.1.3 (R Foundation for Statistical Computing).^33^ Overlap weighting was performed using the PSweight package.^34^ A full list of packages used is given in the eMethods in Supplement 1. Missingness was described for each variable. A complete case analysis was used for all analytic steps.

## Results

### Characteristics

There were 19 915 hospitalizations among 15 072 unique patients aged 18 to 39 years (49.6% male; median age, 30 years; interquartile range [IQR], 24-35 years) in the cohort (eFigure in Supplement 1). Among all hospitalizations, 1329 (6.7%) had 1 or more diagnostic codes indicating 4Cs, representing 814 unique patients (5.4%). Most hospitalizations with 4Cs had only one 4C code (1232 of 1329 [92.7%]). The most common 4C subcategories among first (index) hospitalizations (814 individuals) were hereditary anemias (212 [26.0%]), cystic fibrosis (138 [17.0%]), cerebral palsy (96 [11.8%]), brain and spinal cord malformations (59 [7.2%]), and heart and vessel malformations (57 [7.0%]).

Demographic and clinical characteristics stratified by the primary exposure (4Cs) are summarized in Table 1. Before overlap weighting, a greater proportion of individuals hospitalized with 4Cs (n = 814) vs those without 4Cs (n = 14 258) were in the younger age categories (eg, 18-24 years: 276 [33.9%] vs 3531 [24.8%]) and had lower CCI scores at admission (eg, ≥3: 7 of 757 [0.9%] vs 381 of 13 547 [2.8%]), with the most common comorbidity in both groups being diabetes (46 [5.7%] and 1112 [7.8%], respectively) (Table 2). A greater proportion of patients with 4Cs were admitted to teaching hospitals (453 [55.6%] vs 6094 [42.7%]), and patients with 4Cs also had higher mLAPS (median, 15.0 [IQR, 5.0-24.5] vs 10.0 [IQR, 1.0-21.0]). The most common reasons for admission were sickle cell disease with crisis (146 [17.9%]) and cystic fibrosis with pulmonary manifestations (103 [12.7%]) among those with 4Cs and diabetic ketoacidosis (383 [2.7%]) among those without (Table 3). Grouping by Clinical Classifications Software Refined category did not alter the most common reason for admission in the cohort with 4Cs, but for those without 4Cs, the most common reason for admission was drug poisoning (855 [6.0%]) (eTable 1 in Supplement 1).

**Table 1.** Characteristics of Young Adults With and Without 4Cs at Index Hospitalization Before and After Weighting

Characteristic	Before weighting			After weighting^b^		
	Patients, No. (%)		SMD^a^	Patients, %		SMD
	With 4Cs (n = 814)	Without 4Cs (n = 14 258)		With 4Cs (n = 802)	Without 4Cs (n = 13 824)	
Age group, y						
18-24	276 (33.9)	3531 (24.8)	0.24	33.0	33.1	0.00
25-29	200 (24.6)	3168 (22.2)		23.9	23.9	
30-39	338 (41.5)	7559 (53.0)		43.0	43.0	
Sex						
Female	Suppressed^c^	Suppressed^c^	0.01	Suppressed^c^	Suppressed^c^	0.00
Male	401 (49.3)	7073 (49.6)		49.3	49.3	
Other or unknown	0	Suppressed^c^		NA	NA	
Income quintile						
First (lowest)_	207 (25.8)	3909 (28.3)	0.07	26.1	26.1	0.00
Second	167 (20.8)	2943 (21.3)		20.8	20.8	
Third	156 (19.4)	2705 (19.6)		19.4	19.4	
Fourth	145 (18.1)	2273 (16.4)		17.8	17.8	
Fifth (highest)	127 (15.8)	1997 (14.4)		15.9	15.9	
Missing	12 (1.5)	431 (3.0)		NA	NA	
Charlson Comorbidity Index score at admission		13547				
0	660 (87.2)	11 553 (85.3)	0.14	87.2	84.3	0.17
1	50 (6.6)	867 (6.4)		6.7	6.4	
2	40 (5.3)	746 (5.5)		5.2	6.1	
≥3	7 (0.9)	381 (2.8)		0.9	3.2	
Missing	57 (7.0)	711 (5.0)		6.8	5.6	
mLAPS						
Median (IQR)	15.0 (5.0-24.5)	10.0 (1.0-21.0)	0.16	15.0 (5.0-25.0)	8.0 (1.0-20.0)	0.22
Missing	182 (22.4)	2589 (18.2)		178 (22.2)	2522 (18.2)	
Teaching hospital	453 (55.6)	6094 (42.7)	0.26	53.4	52.7	0.01

Abbreviations: 4Cs, childhood-onset complex chronic conditions; mLAPS, modified Laboratory Acute Physiology Score; NA, not applicable; SMD, standardized mean difference.

^a^ The percentages and SMDs were calculated without the missing cases.

^b^ Weighted using a propensity score model that only included the following baseline characteristics and a complete case analysis: age group, sex, neighborhood income quintile, and hospital number as a random effect. The rest of the characteristics are for descriptive purposes. Other or unknown values for sex and missing values for neighborhood income were not included in the propensity score model to perform the overlap weighting and, thus, were not included in the postweighting percentages or in the calculation of standardized differences for sex and income quintile before and after weighting.

^c^ Values less than 6 were suppressed for patient privacy. Data for females were suppressed due to possibility of back-calculating from male and other or missing categories.

**Table 2.** Five Most Common Charlson Comorbidities During Index Hospitalizations Among Patients With and Without 4Cs^a^

Comorbidity	Patients, No. (%)
4Cs (n = 814)	
Diabetes	46 (5.7)
Kidney disease	37 (4.5)
Chronic lung disease	27 (3.3)
Mild liver disease	24 (2.9)
Cancer	18 (2.2)
No 4Cs (n = 14 258)	
Diabetes	1112 (7.8)
Cancer	646 (4.5)
Chronic lung disease	432 (3.0)
Mild liver disease	428 (3.0)
Diabetes with complications	390 (2.7)

Abbreviation: 4Cs, childhood-onset complex chronic conditions.

^a^ Disease categories, including definitions of mild vs moderate or severe liver disease, were defined as by Quan et al.^21^ Charlson comorbidities were tabulated without duplication of the same condition in the same individual using inpatient diagnostic codes and regardless of the condition’s status as a comorbidity with reference to the reason for admission. Thus, the numbers reported are slightly higher (but with no difference in the most common diagnoses) for each chronic condition compared with the Charlson Comorbidity Index score derived based on the reason for admission and emergency department diagnoses.

**Table 3.** Five Most Common Reasons for Index Hospitalizations Among Patients With and Without 4Cs^a^

Diagnosis	Patients, No. (%)
4Cs (n = 814)	
Sickle cell disease with crisis	146 (17.9)
Cystic fibrosis with pulmonary manifestations	103 (12.7)
Aspiration pneumonitis	27 (3.3)
Pneumonia	24 (2.9)
Other and unspecified convulsions	9 (1.1)
No 4Cs (n = 14 258)	
Diabetic ketoacidosis	383 (2.7)
Alcohol withdrawal	295 (2.1)
Pneumonia	206 (1.4)
Acetaminophen overdose	199 (1.4)
Gastroenteritis	167 (1.2)

Abbreviation: 4Cs, childhood-onset complex chronic conditions.

^a^ Limited to 1 diagnosis per individual if there were multiple diagnoses (about 10 total).

After applying overlap weighting, age, sex, and income were balanced between groups (Table 1). The proportion of patients admitted to each of the 29 hospitals was balanced as well, with similar proportions being admitted to teaching hospitals after weighting. The CCI scores and mLAPS, which were not included in the propensity score, were similar to those before weighting.

### Outcomes

Hospitalizations with 4Cs accounted for 13 606 of 126 910 total adult hospital bed-days (10.7%). The mean (SD) number of care episodes in the cohort with 4Cs was 1.51 (1.20) compared with 1.28 (0.94) for comparators (SMD = 0.22). Outcomes before and after weighting at the patient level are shown in Table 4. After weighting, index hospitalizations with 4Cs were associated with longer length of stay (relative ratio [RR], 1.62; 95% CI, 1.48-1.77). There were no differences in the relative rate of in-hospital mortality (1.43; 95% CI, 0.87-2.33) or ICU admissions (1.05; 95% CI, 0.91-1.20) among hospitalizations with and without 4Cs. For secondary outcomes, hospitalizations with 4Cs were associated with higher total hospital costs (RR, 1.65; 95% CI, 1.05-2.59), greater number of medication orders (RR, 1.26; 95% CI, 1.19-1.34), fewer advanced imaging orders (RR, 0.85; 95% CI, 0.77-0.93), and higher 30-day readmission rates (RR, 1.59; 95% CI, 1.28-1.98).

**Table 4.** Outcomes of Index Hospitalizations Among Young Adults With and Without 4Cs Before and After Weighting

Outcome	Before weighting		After weighting		
	Patients with 4Cs (n = 814)^a^	Patients without 4Cs (n = 14 258)^a^	Patients with 4Cs (n = 802)^b^	Patients without 4Cs (n = 13 824)^b^	Relative outcome ratio (95% CI)^c^
**Primary outcomes**					
ICU admissions	179 (22.0)	2922 (20.5)	22.5	21.5	1.05 (0.91-1.20)
In-hospital mortality	19 (2.3)	213 (1.5)	2.3	1.6	1.43 (0.87-2.33)
Length of stay, median (IQR), d	4.7 (2.2-9.4)	2.6 (1.2-5.4)	4.7 (2.2-9.4)	2.7 (1.3-5.6)	1.62 (1.48-1.77)
**Secondary outcomes**					
Total cost					
Median (IQR), $	6474 (3815-15 092)	4313 (3089-7871)	6274 (3786-15 092)	4350 (3177-8265)	1.65 (1.05-2.59)
Missing	144 (17.7)	1177 (8.3)	143 (17.8)	1110 (8.0)	
30-d readmissions^d^					
Value	88 (11.4)	966 (7.1)	11.2	7.1	1.59 (1.28-1.98)
Missing^a^	39 (4.8)	659 (4.6)	4.8	4.5	
Medications					
Median (IQR)	14 (9-21)	10 (6-16)	14 (9-21)	10 (6-17)	1.26 (1.19-1.34)
Missing	148 (18.2)	3628 (25.4)	147 (18.3)	3515 (25.4)	
Advanced imaging scans					
0	490 (60.2)	8382 (58.8)	60.1	55.5	0.84 (0.77-0.93)^e^
1	198 (24.3)	3292 (23.1)	24.4	23.5	
≥2	126 (15.5)	2584 (18.1)	15.5	21.0	

Abbreviations: 4Cs, childhood-onset complex chronic conditions; ICU, intensive care unit.

^a^ Data are presented as number (percentage) of patients unless otherwise indicated.

^b^ Data are presented as weighted percentage of patients unless otherwise indicated.

^c^ ICU admission, in-hospital mortality, and 30-day readmissions are risk ratios, length of stay and costs are relative ratios, and medications and advanced imaging are rate ratios.

^d^ Thirty-day readmission was coded as not applicable if death occurred in hospital or if transfer to another acute care hospital occurred given that length of time admitted to the transferred hospital was unknown and could not be assumed.

^e^ The relative rate was adjusted after excluding 4 hospitals without radiology data.

### Sensitivity Analyses

When expanding the cohort definition of 4Cs to include possibly pediatric-onset conditions, 4Cs made up 29% (4446) of all patients and 55.3% (70 220) of young adult hospital bed-days. The RRs of each outcome were similar to those observed in the primary analysis (eResults and eTables 2-6 in Supplement 1). Restricting analysis to patients aged 18 to 24 years yielded results consistent with those of the primary analysis in terms of characteristics, outcome associations, and the most common diagnoses (eTables 7-10 in Supplement 1).

## Discussion

In this cohort study, we adapted a widely used algorithm to identify 4Cs in adult hospitalization data, observing that young adults with 4Cs represented 6.7% of young adult hospitalizations. Consistent with our hypothesis, we found that this cohort had longer hospital stays, higher hospitalization costs, and higher risk of readmission within 30 days than other young adults admitted to acute care medical centers. These results were robust to a sensitivity analysis using a broader cohort definition of 4Cs. The findings provide data on the impacts of 4Cs in adult inpatient medicine within a universal health care system in which there is mandatory transfer to adult care for all individuals older than 18 years and highlight opportunities for targeted interventions to improve care.

Our results are similar to those from a US study that indicated higher inpatient resource use for adults with pediatric-onset disabilities.^11^ We expand on these results by (1) including 4Cs in our cohort definition that are not captured under congenital malformation categories in the *ICD-10*, including sickle cell disease and cystic fibrosis (the most common 4Cs identified in the current study)^11^; (2) moving closer to assessing system-level impacts of 4Cs by using a standardized adult care setting in a population with universal health care; and (3) providing specific outcome measures, including length of stay, 30-day readmission rates, and total costs, all of which are potential targets for acute care interventions. The similarity of our findings to the US study^11^ despite differing cohort definitions suggests that there may be disease-independent effects of 4Cs on adult medicine across different health care systems.

Our results showed that a substantial proportion of admissions among patients with 4Cs were related to the 2 most common conditions (17.9% for sickle cell disease; 12.7% for cystic fibrosis with pulmonary manifestations), highlighting an opportunity to refine existing targeted outpatient interventions^35,36,37,38^ for these conditions to help reduce length of stay and readmissions.^35,36^ For example, recent efforts have shown success in these metrics for sickle cell disease by implementing adult centers of expertise and through medical education interventions.^36^ Better integration of outpatient and inpatient care may help reduce length of stay and prevent readmissions. There may be components of such disease-specific interventions that could be applicable to the broader population with 4Cs.

It is possible that part of the observed greater hospitalization resource use was related to known service disruption and/or fragmentation at the point of transition to adult care.^39,40^ Studies focusing only on the first 1 to 2 years after age 18 years have not indicated increased resource use but would not capture the full and lasting effects of transition.^1,8^ In contrast to focusing on transition as an event, the current study examined posttransition outcomes in young adults aged 18 to 39 years. The findings suggest that extending pediatric models of care^5,41,42^ to adult medicine^43^ could also help improve quality of care and reduce hospital resource use in young adulthood. For example, a 4C clinic mirroring the structure of pediatric complex care clinics and led by general internists and/or hospitalists could help assess and address challenges in adult system navigation. A unified inpatient consultation service could help reduce long lengths of stay and delays in care that may be related to lack of familiarity of the condition or challenges accessing necessary advanced imaging^41^; less advanced imaging tests may be related to challenges in obtaining them, leading to longer lengths of stay. Also, longitudinal outpatient care models may facilitate management of evolving complexity with aging, as is the case for specific complex conditions.^44,45,46^ A hospital-associated outpatient referral pathway for these patients may also lead to streamlining inpatient-to-outpatient care transitions, improving navigation through the adult system and thereby mitigating the risk of preventable medical admissions and readmissions.^47^

Better appreciation of the mechanisms leading to greater illness severity could help in developing specific interventions to improve care. Notably, patients with 4Cs had lower CCI scores than their comparators despite increased health care utilization, suggesting that this index has limited utility in capturing complexity and predicting outcomes in young adults. Developing a valid comorbidity index for younger adults may be valuable in risk stratifying those at highest risk of poor outcomes.

### Strengths and Limitations

Strengths of our study include adaptation of a widely used algorithm^1,3^ to identify a group with 4Cs within a well-defined adult medical care system and findings robust to sensitivity analyses. The GEMINI dataset enabled us to assess many aspects of inpatient care using detailed electronic health data, similar to what would be available in other centers.

Our study also has several limitations. While the CCC system is commonly used, other classification systems exist that would not identify the same group of patients.^48^ Although adaptation of the CCC algorithm and classification of a pediatric-onset condition was conducted by content experts, other experts may have made different classification decisions. The sensitivity and specificity of the CCC algorithm depends on component *ICD-10* codes and may lack accuracy for some diagnoses, such as those representing rare diseases.^49^ While the hospital-based GEMINI dataset allowed us to follow up patients into early adulthood, only adult data were available; thus, we were unable to look back to childhood for age at diagnoses, which would have helped optimize the cohort definition and will be necessary for future validation studies. GEMINI covers about 30 hospitals in diverse geographic settings across Ontario, including more remote areas; however, these are mainly large urban or suburban hospitals and disproportionately include tertiary care centers in the greater Toronto area. GEMINI data do not include surgical, obstetrical, psychiatric, or emergency department–only admissions; thus, findings may not reflect the impacts of 4Cs in nonmedical hospital inpatient settings. Data were restricted to general medicine admissions at 4 hospitals during the period studied, potentially resulting in undersampling of admissions for certain subgroups with 4Cs (eg, adults with congenital cardiac disease admitted to the cardiology unit, although the majority of admissions for congenital heart disease tend to occur in adults older than 40 years^50^). By choosing to focus on an index admission, we may have missed important patterns of repeated admissions. There were also some missing data for several secondary outcomes. We could not account for out-of-hospital deaths or transfers, and this may have introduced bias in assessing 30-day readmission rates. However, more than 80% of Ontario hospital readmissions are reported to occur at the same hospital.^51^ Linkage to population-based datasets will be important to validate our CCC classification algorithm and our study findings.

## Conclusions

In this cohort study of young adults discharged from 29 hospitals, individuals within a group defined by 4Cs had longer acute care medical hospitalizations and more rehospitalizations than other young adults. The results suggest that this resource-intensive population should be prioritized for targeted policy interventions to reduce inpatient stays and improve care. The CCI may be of limited utility for capturing comorbidities in this population.

## Notes

Supplement 1.
eAppendix.

eMethods.
**eResults.** Sensitivity analysis **eFigure.** Cohort creation **eTable 1.** Top 5 most responsible diagnoses for index hospitalizations (individuals) in childhood-onset complex chronic condition (4C) cohort and comparator groups, by CCSR category **eTable 2.** Baseline characteristics stratified by childhood-onset complex chronic conditions (4Cs) for all young adult (<40 years old) index hospitalizations in the GEMINI cohort in 2018 before and after weighting **eTable 3.** Top 5 most common Charlson comorbidities for index hospitalizations with and without 4Cs **eTable 4.** Top 5 most responsible diagnoses for index hospitalizations with and without 4Cs **eTable 5.** Top 5 most responsible diagnoses for index hospitalizations with and without 4Cs, grouped by CCSR category **eTable 6.** Outcomes stratified by childhood-onset complex chronic conditions (4Cs) for all young adult (<40 years) index hospitalizations in the GEMINI cohort in 2018 before and after weighting **eTable 7.** Baseline characteristics stratified by childhood-onset complex chronic conditions (4Cs) for young adults index hospitalizations in the GEMINI cohort in 2018 before and after weighting: restricted to age 18-24 years **eTable 8.** Top 5 most common Charlson comorbidities for index hospitalizations with and without 4Cs, 18-24 year olds **eTable 9.** Top 5 most responsible diagnoses for index hospitalizations with and without 4Cs, 18-24 year olds **eTable 10.** Outcomes stratified by childhood-onset complex chronic conditions (4Cs) for young adult index hospitalizations in the GEMINI cohort in 2018 before and after weighting, restricted to age 18-24 years
eReferences.

Supplement 2. **eTable.** GEMINI code description

Supplement 3.
Data Sharing Statement
