# Supplementary material for: Characteristics and Outcomes of Adults Hospitalized With Childhood-Onset Complex Chronic Conditions
Source: JAMA Netw Open. 2026 Jan 28;9(1):e2553610. doi: 10.1001/jamanetworkopen.2025.53610 (PMC12853208; doi:10.1001/jamanetworkopen.2025.53610)
Supplement: Supplement 1. — eAppendix. eMethods. eResults. Sensitivity analysis eFigure. Cohort creation eTable 1. Top 5 most responsible diagnoses for index hospitalizations (individuals) in childhood-onset complex chronic condition (4C) cohort and comparator groups, by CCSR category eTable 2. Baseline characteristics stratified by childhood-onset complex chronic conditions (4Cs) for all young adult (<40 years old) index hospitalizations in the GEMINI cohort in 2018 before and after weighting eTable 3. Top 5 most common Charlson comorbidities for index hospitalizations with and without 4Cs eTable 4. Top 5 most responsible diagnoses for index hospitalizations with and without 4Cs eTable 5. Top 5 most responsible diagnoses for index hospitalizations with and without 4Cs, grouped by CCSR category eTable 6. Outcomes stratified by childhood-onset complex chronic conditions (4Cs) for all young adult (<40 years) index hospitalizations in the GEMINI cohort in 2018 before and after weighting eTable 7. Baseline characteristics stratified by childhood-onset complex chronic conditions (4Cs) for young adults index hospitalizations in the GEMINI cohort in 2018 before and after weighting: restricted to age 18-24 years eTable 8. Top 5 most common Charlson comorbidities for index hospitalizations with and without 4Cs, 18-24 year olds eTable 9. Top 5 most responsible diagnoses for index hospitalizations with and without 4Cs, 18-24 year olds eTable 10. Outcomes stratified by childhood-onset complex chronic conditions (4Cs) for young adult index hospitalizations in the GEMINI cohort in 2018 before and after weighting, restricted to age 18-24 years eReferences. [file jamanetwopen-e2553610-s001.pdf]

Supplemental Online Content

Malecki SL, Shen T, Loffler A, et al. Characteristics and outcomes of adults hospitalized with childhood-onset complex chronic conditions. *JAMA Netw Open*. 2026;9(1):e2553610. doi:10.1001/jamanetworkopen.2025.53610

eAppendix.
eMethods.
eResults. Sensitivity analysis.
eFigure. Cohort creation.
eTable 1. Top 5 most responsible diagnoses for index hospitalizations (individuals) in childhood-onset complex chronic condition (4C) cohort and comparator groups, by CCSR category.
eTable 2. Baseline characteristics stratified by childhood-onset complex chronic conditions (4Cs) for all young adult (<40 years old) index hospitalizations in the GEMINI cohort in 2018 before and after weighting.
eTable 3. Top 5 most common Charlson comorbidities for index hospitalizations with and without 4Cs.
eTable 4. Top 5 most responsible diagnoses for index hospitalizations with and without 4Cs.
eTable 5. Top 5 most responsible diagnoses for index hospitalizations with and without 4Cs, grouped by CCSR category.
eTable 6. Outcomes stratified by childhood-onset complex chronic conditions (4Cs) for all young adult (<40 years) index hospitalizations in the GEMINI cohort in 2018 before and after weighting.
eTable 7. Baseline characteristics stratified by childhood-onset complex chronic conditions (4Cs) for young adults index hospitalizations in the GEMINI cohort in 2018 before and after weighting: restricted to age 18-24 years.
eTable 8. Top 5 most common Charlson comorbidities for index hospitalizations with and without 4Cs, 18-24 year olds.
eTable 9. Top 5 most responsible diagnoses for index hospitalizations with and without 4Cs, 18-24 year olds.
eTable 10. Outcomes stratified by childhood-onset complex chronic conditions (4Cs) for young adult index hospitalizations in the GEMINI cohort in 2018 before and after weighting, restricted to age 18-24 years.
eReferences.

This supplemental material has been provided by the authors to give readers additional information about their work.

eAppendix

See separate excel file entitled "eAppendix-CCC_gemini_code_descriptions_May 28 2025". This file contains the full list of ICD-10 codes in the CCC algorithm, their descriptions, and the pediatric categorization to apply to adult data. Codes were searched in hospital data by pattern matching at the start of the string using the grpl function. ICD-10 code descriptions were obtained on the Canadian Institutes for Health Information (CIHI) e-store (under Category/Rubric Tables: ICD-10-CA). Special conditions (e.g. X not Y) were coded manually and descriptions were not matched for these.

eMethods

Cohort and comparator

We adapted the pediatric "CCC" algorithm initially developed by Feudtner et al in 2000[1] and updated in 2014.[2] The algorithm is a compiled list of *International Statistical Classification of Diseases and Related Health Problems 10th Revision* (ICD-10) and procedure codes, each indicating the presence of a CCC. The algorithm was developed and validated in US pediatric hospital data which uses ICD-10-CM codes. Version 3 was released in 2024.[3] Version 2 (2014) was applied here due to its widespread use in pediatric health service research, including translating the US codes in the algorithm to Canadian codes (ICD-10-CA) for use in Canadian pediatric data by the Canadian Institutes for Health Information (CIHI),[4,5] as detailed in the main text. Procedure codes in the original CIHI adaptation of the CCC algorithm[5] were not used or adapted for this study given they are typically related to body parts rather than conditions, and their relation to childhood would be difficult to determine. ICD-10 codes were grouped into body system categories, as in the original algorithm.[2,4] Classifications were further refined after preliminary inspection of the prevalence of codes in each cohort, e.g. codes for lipid disorders, electrolyte abnormalities and cardiomyopathy were moved out of the "most likely pediatric" category, given their high prevalence in the adult cohort and their likely use to indicate common adult onset conditions rather than rarer pediatric-onset conditions. Codes Q21.8, Q21.9, Q22, Q23, Q24 were added and classified as "most likely pediatric" after confirming with CIHI that they had been unintentionally excluded during the adaptation of the US algorithm to Canadian data.

Demographic and clinical characteristics

Neighborhood-level income quintiles were obtained by linking patients' postal codes to Statistics Canada's 2016 census data at the level of dissemination areas, using the Postal Code Conversion File Plus program.[6] The mlaps score was chosen as a measure of illness severity. This score is frequently used in Gemini as a valid index of illness severity[7] over other indices used in clinical practice, due to the ready availability of these laboratory parameters in the GEMINI dataset, and the inconsistent availability of other predictors included in other clinical indices, e.g. vital signs, in hospital administrative data.

Number of 4Cs: The counts represent unique diagnostic code entries into the hospital discharge abstract database; these may be different codes indicating complications of the same underlying diagnosis eg cancer, or may be the same code repeated for different diagnosis types.

Estimating Total inpatient cost

1. Used CSHS (cost per standard hospital stay), which is CIHI information generally within the public domain.
2. Formula for total cost = Resource Intensity Weighting (RIW) * [Cost Per Weighted Case (CPWC) or Cost of a Standard Hospital Stay (CSHS)].

Resources:
CIHI CSHS**:** https://yourhealthsystem.cihi.ca/hsp/inbrief?lang=en#!/indicators/015/cost-of-a-standard-hospital-stay-cshs/;mapC1;mapLevel2;provinceC9001;trend(C1,C5001);/
Ontario case costing methodology**:** https://tspace.library.utoronto.ca/bitstream/1807/87373/1/Wodchis%20et%20al_2013_Guidelines%20on%20Person-Level%20Costing.pdf
Stats Canada CPI inflation factors**:** https://www150.statcan.gc.ca/t1/tbl1/en/tv.action?pid=1810000501

Hospital data for this study was from 2018 Calander year, corresponding mainly to 2017 and 2018 fiscal years. Four hospitals also provided their RIW data using 2020 and 2021 methodology years. We obtained 2017, 2018, 2020 and 2021 CSHS from CIHI (either through their website, or from CIHI directly for 2017 data (suppressed) since it was no longer available publicly. We used Ontario-specific CSHS since hospital data is from Ontario and chose provincial level estimates over hospital-specific estimates as this minimizes effect of region on cost.

CSHS by methodology year:
2017 – suppressed
2018 - $5741
2020 - $6767
2021 - $6630

We multiplied RIW x CSHS (for that methodology year) for each row to get total cost for each encounter and subsequently inflated the value to 2023 dollars by multiplying costs by the yearly consumer price index inflation factors (CPI) provided by Statistics Canada (yearly for health and personal care in Ontario):

For example, for 2017, factor will be CPI 2023/CPI 2017

CPI by year:
2017 - 125.8
2018 - 128.0
2020 - 129.6
2021 - 133.3
2023 - 147.6

Given that hospitals provided data from different methodology years, we first performed sensitivity analyses to ensure there were no significant differences in costs related to this. Three cost variables were created after:

1 - Eliminating the 4 hospitals that didn't provide the correct methodology year (used 2020, 2021 years)
2 - Eliminating all data from the 2017 fiscal year
3 - 1 and 2

We then used independent sample t-tests to compare the means and Wilcoxon rank sum tests to compare the medians of the original cost variable versus costs calculated these 3 ways. We used a p-value cutoff of <0.05 to determine significance. After confirming there were no significant differences, all data were combined in a single cost variable for the cohort.

Counting number of medications

We used the first 5 characters of the generic name for each row within each encounter to count medication orders. We standardized text to lowercase and extracted the first 5 characters. Number of medications was inputted as 0 if there were no medication records but there was pharmacy data available for that hospital during the time period.

List of packages used and different steps:

1. Cohort creation:
   rstudioapi
   dplyr
   readxl
   data.table
   DBI
   RPostgreSQL
   ggplot2
   lubridate

2. Baseline characteristics, qualitative categories
   Tableone
   Survey
   Stringr
   Rgemini

3. Overlap weighting
   lme4
   PSweight
   lmerTest

4. Weighted regression
   nortest
   lmtest
   sandwich

eResults

Sensitivity analysis

Our sensitivity analysis, described as well in the main text, expanded the cohort definition of the childhood-onset complex disease (4C) cohort to include "possibly pediatric" onset conditions is shown in eTables 2-6. 6611 (33.2%) of encounters had at least one diagnostic code indicating 4Cs, representing (4446, 29%) of unique patients. Number of 4C codes ranged from 1-12, with a median of one condition (IQR 1-2). Using this expanded definition, the most common 4C subcategories among individuals were neoplasms (n=885, 20%), devices (n=695, 16%), inflammatory bowel diseases (n=581, 13%), chronic respiratory diseases (n=439, 10%) and chronic renal failure (n=226, 5%). The major difference in baseline characteristics compared to our primary analysis was that CCI scores were higher among individuals with 4Cs (eTable 3), with the most common Charlson condition being cancer (eTable 3). The top reason for admission among both groups did not change (eTable 4), except when grouping by CCSR category (eTable 5), which resulted in ulcerative colitis and poisoning by drugs being the most common reasons for admission among those with and without 4Cs respectively. 4Cs made up 70220.47 (55.3%) of adult hospital bed days. After accounting for age, sex, neighborhood income and hospital effects, the relative risk/rate/ratio of 4Cs on each outcome was significantly greater than 1, demonstrating consistency with our primary analysis except for the number of computed tomography (CT)s and magnetic resonance imaging (MRIs), which were lower among those with 4Cs in the primary analysis (eTable 6). Increasing the sensitivity of our cohort definition also resulted in a significant effect of 4Cs on in-hospital mortality (RR 5.72, 95% CI 4.23-7.74) and ICU admission (RR 1.30, 95% CI 1.21-1.38), with the former being the most pronounced effect of all the outcomes (eTable 6).

eFigure 1. Cohort Creation.

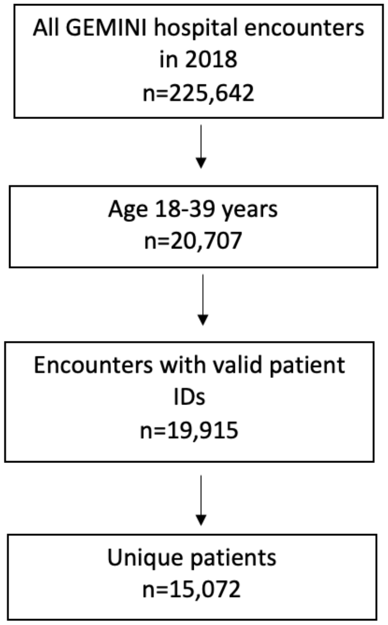

eTable 1. Top 5 most responsible diagnoses for index hospitalizations (individuals) in childhood-onset complex chronic condition (4C) cohort and comparator groups, by CCSR category[a]

4Cs (N= 814)	n (%)	No 4Cs (N= 14258)	n (%)
BLD005 Sickle cell trait/anemia	150 (18.4)	INJ022 Poisoning by drugs	855 (6.0)
END012 Cystic fibrosis	116 (14.3)	END003 Diabetes with complication	593 (4.2)
RSP002 Pneumonia	46 (5.7)	NVS009 Epilepsy/convulsions	569 (4.0)
NVS009 Epilepsy/convulsions	38 (4.7)	DIG001 Intestinal infection	401 (2.8)
RSP010 Aspiration pneumonitis	27 (3.3)	MBD017 ETOH-related disorders	401 (2.8)

[a] The same category could not be repeated per individual.

eTable 2. Baseline characteristics stratified by childhood-onset complex chronic conditions (4Cs) for all young adult (<40 years old) index hospitalizations in the GEMINI cohort in 2018 before and after weighting.

	Before weighting, N (%)			After weighting,[c] %		
**Characteristic**	**4Cs[a]**	**No 4Cs**	**SMD[b]**	**4Cs**	**No 4Cs**	**SMD**
**N**	4446	10626				
**Age group**			0.03			0.0
**18-24**	1091 (24.5)	2716 (25.6)		24.8	24.8	
**25-29**	977 (22.0)	2391 (22.5)		21.9	21.9	
**30-39**	2378 (53.5)	5519 (51.9)		53.3	53.3	
**Sex**						
**Male**	2263 (50.9)	5211 (49.1)	0.04	50.4	50.4	0.0
**Unknown/missing**	0 (0.0)	Suppressed[d]		NA	NA	
**Income quintile**			0.07			0.0
**1**	1172 (26.9)	2944 (28.6)		27.5	27.5	
**2**	906 (20.8)	2204 (21.4)		21.1	21.1	
**3**	823 (18.9)	2038 (19.8)		19.0	19.0	
**4**	781 (17.9)	1637 (15.9)		17.2	17.2	
**5**	669 (15.4)	1455 (14.2)		15.1	15.1	
**Missing**	95 (2.1)	348 (3.3)		NA	NA	
**Admission Charlson score**			0.58			0.56
**0**	2897 (71.3)	9316 (91.0)		71.5	90.8	
**1**	326 (8.0)	591 (5.8)		8.3	5.7	
**2**	542 (13.3)	244 (2.4)		12.9	2.5	
**3+**	300 (7.4)	88 (0.9)		7.2	1.0	
**Missing**	381 (8.6)	387 (3.6)		8.3	4.2	
**Mlaps (Median [IQR])**	12 [5-25]	7 [1-20]	0.25	13 [5-25]	8 [1-20]	0.23
**Missing**	1163 (26.0)	1108 (10.4)		1138 (26.2)	1562 (15.2)	
**Teaching hospital**	2416 (54.3)	4131 (38.9)	0.31	48.7	48.0	0.01

SMD = standardized mean differences, IQR= interquartile range. Mlaps = modified laboratory acute physiology score (see text for further description).[a] Sensitive definition of 4Cs, including individuals with most likely pediatric codes or possibly pediatric codes (see text for further details).
[b] The standardized differences were calculated without the missing cases.
[c] Note that the propensity score model (outcome = 4Cs) only includes the following baseline characteristics as predictors and uses a complete case analysis: age group, sex, neighborhood income quintile, hospital number as a random effect. The rest of the characteristics displayed here are for descriptive purposes. Note after weighting N=14626, 4351 in the 4Cs group and 10275 in the no 4Cs group. Other/unknown values for sex and missing values for neighbourhood income were not included in the propensity score model to perform the overlap weighting so were not included in the post weighting percentages or in the calculation of standardized differences for sex, and income quintile before and after weighting.
[d] Values less than 6 are suppressed to avoid potential individual patient identification.

eTable 3. Top 5 most common Charlson comorbidities for index hospitalizations with and without 4Cs[a,b]

4Cs (N=4446)	N (%)	No 4Cs (N=10626)	N (%)
Cancer	653 (14.7)	Diabetes	855 (8.0)
Diabetes	303 (6.8)	Chronic lung disease	347 (3.3)
Renal disease	256 (5.8)	Diabetes with complications	273 (2.6)
Metastatic cancer	206 (4.6)	Mild liver disease	272 (2.6)
Heart failure	187 (4.2)	Cerebrovascular disease	151 (1.4)

[a] Sensitive definition of 4Cs, including individuals with most likely pediatric codes or possibly pediatric codes (see text for further details).
[b] Charlson conditions are tabulated without duplication of the same condition in the same individual, and regardless of the condition's status as a comorbidity with reference to the most responsible diagnosis, which is different from how the overall admission Charlson comorbidity score is calculated.

eTable 4. Top 5 most responsible diagnoses for index hospitalizations with and without 4Cs[a,b]

4Cs (N=4446)	N (%)	No 4Cs (N=10626)	N (%)
Sickle-cell anemia with crisis	146 (3.3)	Diabetic ketoacidosis	301 (2.8)
Ulcerative colitis	120 (2.7)	Alcohol withdrawal	275 (2.6)
Pulmonary embolism	117 (2.6)	Tylenol overdose	192 (1.8)
Cystic fibrosis with pulmonary manifestations	103 (2.3)	Pneumonia	169 (1.6)
Diabetic ketoacidosis	89 (2.0)	Cellulitis	144 (1.4)

[a] Sensitive definition of 4Cs, including individuals with most likely pediatric codes or possibly pediatric codes (see text for further details).
[b]Limited to one most responsible diagnosis per individual, if there were multiple.

eTable 5. Top 5 most responsible diagnoses for index hospitalizations with and without 4Cs, grouped by CCSR category[a,b]

4Cs (N=4446)	N (%)	No 4Cs (N=10626)	N (%)
DIG011 Ulcerative colitis	377 (8.5)	INJ022 Poisoning by drugs	797 (7.5)
NVS009 Epilepsy/convulsions	230 (5.2)	END003 Diabetes with complication	469 (4.4)
BLD005 Sickle cell trait/anemia	150 (3.4)	NVS009 Epilepsy/convulsions	377 (3.5)
END003 Diabetes with complication	133 (3.0)	MBD017 ETOH-related disorders	372 (3.5)
CIR017 Cardiac dysrhythmias	125 (2.8)	DIG020 Pancreatic disorders (excluding diabetes)	351 (3.3)

[a] Sensitive definition of 4Cs, including individuals with most likely pediatric codes or possibly pediatric codes (see text for further details).
[b] The same category could not be repeated per individual.

eTable 6. Outcomes stratified by childhood-onset complex chronic conditions (4Cs) for all young adult (<40 years) index hospitalizations in the GEMINI cohort in 2018 before and after weighting[a]

	Before weighting, N (%)		After weighting, %		
Outcome	4Cs N=4446	No 4Cs N=10626	4Cs N=4351	No 4Cs N=10275	Relative outcome ratio (95% CI)
ICU admission	1173 (26.4)	1928 (18.1)	26.3	20.3	1.30 (1.21-1.38)
In-hospital mortality	169 (3.8)	63 (0.6)	3.7	0.7	5.72 (4.23-7.74)
Length of stay, days (Median [IQR])	4.2 [1.9-9.2]	2.2 [1.1-4.6]	4.2 [1.9-9.0]	2.4 [1.2-4.7]	1.84 (1.76-1.92)
30-day readmission	399 (9.8)	655 (6.3)	9.8	6.2	1.58 (1.40-1.79)
Missing[b]	391 (8.8)	307 (2.9)	8.8	3.1	
Total cost $ (Median[IQR])	6782.21 [3935.88-15092.03]	4041.08 [2944.96-6427.38]	6782.21 [3934.61-14609.73]	4182.78 [3006.52-6751.76]	2.45 (2.14-2.81)
Missing	597 (13.4)	724 (6.8)	578 (13.3)	675 (6.6)	
Number of medications (Median[IQR])	13 [8-21]	9 [6-15]	13 [8-21]	9 [6-15]	1.37 (1.32-1.41)
Missing	1072 (24.1)	2704 (25.4)	1049 (24.1)	2613 (25.4)	
CT or MRI					1.18 (1.13-1.24)
0	2347 (52.8)	6525 (61.4)	53.6	59.8	
1	1072 (24.1)	2418 (22.8)	24.3	22.5	
2+	1027 (23.1)	1683 (15.8)	22.1	17.7	

[a]Sensitive definition of 4Cs, including individuals with most likely pediatric codes or possibly pediatric codes (see text for further details).
[b]30-day readmission was coded as NA if death occurred in hospital, or if transfer to another acute care hospital occurred, given that length of time admitted to the transferred hospital was unknown and could not be assumed.

eTable 7. Baseline characteristics stratified by childhood-onset complex chronic conditions (4Cs) for young adults index hospitalizations in the GEMINI cohort in 2018 before and after weighting: restricted to age 18-24 years.

	Before weighting, N(%)			After weighting,[c] %		
**Characteristic**	**4Cs[a]**	**No 4Cs**	**SMD[b]**	**4Cs**	**No 4Cs**	**SMD**
**N**	276	3531				
**Age group**			0.15			0.0
**18-19**	85 (30.8)	873 (24.7)		30.3	30.4	
**20-22**	107 (38.8)	1565 (44.3)		39.5	39.4	
**23-24**	84 (30.4)	1093 (31.0)		30.2	30.2	
**Sex**			0.02			
**Male**	129 (46.7)	1685 (47.7)		46.6	46.6	0.0
**Unknown/missing**	(suppressed)	(Suppressed)[d]		NA	NA	
**Income quintile**			0.08			0.0
**1**	73 (26.4)	885 (25.7)		26.5	26.4	
**2**	(suppressed)	695 (20.2)		19.4	19.4	
**3**	58 (21.0)	655 (19.0)		21.1	21.1	
**4**	40 (14.5)	587 (17.1)		14.6	14.5	
**5**	50 (18.1)	617 (17.9)		18.4	18.5	
**Missing**	(suppressed)	92 (2.6)		NA	NA	
**Admission Charlson score**			0.10			0.17
**0**	232 (89.9)	3042 (90.3)		90.2	89.7	
**1**	17 (6.6)	167 (5.0)		6.7	4.5	
**2**	(suppressed)	119 (3.5)		(suppressed)	4.0	
**3+**	(suppressed)	42 (1.2)		(suppressed)	1.7	
**Missing**	18 (6.5)	161 (4.6)		6.3	5.0	
**Mlaps (Median [IQR])**	11 [0, 23]	7 [1, 20]	0.12	11 [0, 23]	7 [0, 20]	0.16
**Missing**	66 (23.9)	597 (16.9)		65 (23.7)	581 (16.9)	
**Teaching hospital**	164 (59.4)	1510 (42.8)	0.34	44.0	45.7	0.03

SMD = standardized mean differences, IQR= interquartile range. Mlaps = modified laboratory acute physiology score (see text for further description).

[a] Sensitivity analysis, restricting main analysis (those with most likely pediatric codes) to age 18-24 years.
[b]The standardized differences were calculated without the missing cases.
[c]Note that the propensity score model (outcome = 4Cs) only includes the following baseline characteristics as predictors and uses a complete case analysis: age group, sex, neighborhood income quintile, hospital number as a random effect. The rest of the characteristics displayed here are for descriptive purposes. Note after weighting N=3712, 274 in 4C group and 3438 in the no 4C group. Other/unknown values for sex and missing values for neighbourhood income were not included in the propensity score model to perform the overlap weighting so were not included in the post weighting percentages or in the calculation of standardized differences for sex, and income quintile before and after weighting.
[d]Values less than 6 are suppressed to avoid potential individual patient identification.

eTable 8. Top 5 most common Charlson comorbidities for index hospitalizations with and without 4Cs, 18-24 year olds[a]

4Cs (N=276)	N (%)	No 4Cs (N=3531)	N (%)
Diabetes	18 (6.5)	Diabetes	261 (7.4)
Renal disease	13 (4.7)	Chronic lung disease	133 (3.8)
Chronic lung disease	9 (3.2)	Cancer	120 (3.4)
Mild liver disease	7 (2.5)	Mild liver disease	40 (1.1)
Hemiplegia	6 (2.2)	Cerebrovascular disease	33 (0.9)

[a] Charlson conditions are tabulated without duplication of the same condition in the same individual, and regardless of the condition's status as a comorbidity with reference to the most responsible diagnosis, which differs from how the overall admission Charlson comorbidity score is calculated.

eTable 9. Top 5 most responsible diagnoses for index hospitalizations with and without 4Cs, 18-24 year olds[a]

4Cs (N=276)	N (%)	No 4Cs (N=3531)	N (%)
Sickle-cell anemia with crisis	50 (18.1)	Diabetic ketoacidosis	143 (4.0)
Cystic fibrosis with pulmonary manifestations	42 (15.2)	Tylenol overdose	108 (3.1)
Pneumonia, unspecified	12 (4.3)	Poisoning by other antidepressants	58 (1.6)
Aspiration pneumonitis	12 (4.3)	Nausea with vomiting	55 (1.6)
Supressed		Asthma, unspecified	52 (1.5)

[a] Limited to one most responsible diagnosis per individual, if there were multiple.

eTable 10. Outcomes stratified by childhood-onset complex chronic conditions (4Cs) for young adult index hospitalizations in the GEMINI cohort in 2018 before and after weighting, restricted to age 18-24 years.

	Before weighting, N (%)		After weighting, %		
**Outcome**	**4Cs N=276**	**No 4Cs N=3531**	**4Cs N=274**	**No 4Cs N=3438**	**Relative outcome ratio (95% CI)**
**ICU admission**	58 (21.0)	717 (20.3)	21.8	21.7	1.01 (0.79-1.28)
**In-hospital mortality**	9 (3.3)	49 (1.4)	3.2	1.7	1.84 (0.86-3.95)
**Length of stay, days (Median [IQR])**	4.62 [2.00, 9.34]	2.25 [1.12, 4.81]	4.56 [1.99, 9.26]	2.55 [1.26, 5.27]	1.74 (1.50-2.03)
**30-day readmission**	37 (14.0)	202 (6.0)	14.3	5.8	2.45 (1.73-3.48)
**Missing[a]**	12 (4.3)	146 (4.1)	4.3	4.3	
**Total cost $ (Median[IQR])**	6163.30 [3786.28, 16890.65]	4080.84 [2890.97, 6782.21]	5884.65 [3786.28, 16343.41]	4182.78 [3006.52, 7721.71]	1.51 (0.94-2.41)
**Missing**	60 (21.7)	265 (7.5)	60 (21.9)	266 (7.7)	
**Number of medications (Median[IQR])**	14.00 [8.50, 20.00]	9.00 [5.00, 15.00]	13 [8, 20]	9 [5, 16]	1.35 (1.20-1.52)
**Missing**	49 (17.8)	880 (24.9)	49 (17.9)	880 (25.6)	
**CT or MRI**					0.76 (0.63-0.92)
**0**	177 (64.1)	2216 (62.8)	64.0	58.1	
**1**	64 (23.2)	714 (20.2)	23.4	20.1	
**2+**	35 (12.7)	601 (17.0)	12.5	21.7	

[a]30-day readmission was coded as NA if death occurred in hospital, or if transfer to another acute care hospital occurred, given that length of time admitted to the transferred hospital was unknown and could not be assumed.

References

1. Feudtner C, Christakis DA, Connell FA. Pediatric deaths attributable to complex chronic conditions: a population-based study of Washington State, 1980-1997. *Pediatrics.* 2000;106(1 Pt 2):205-209.
2. Feudtner C, Feinstein JA, Zhong W, Hall M, Dai D. Pediatric complex chronic conditions classification system version 2: updated for ICD-10 and complex medical technology dependence and transplantation. *BMC Pediatrics.* 2014;14(1):199.
3. Feinstein JA, Hall M, Davidson A, Feudtner C. Pediatric Complex Chronic Condition System Version 3. *JAMA Network Open.* 2024;7(7):e2420579-e2420579.
4. Canadian Institute for Health Information. Children and youth with medical complexity in Canada. https://www.cihi.ca/en/children-and-youth-with-medical-complexity-in-canada. Accessed 17 May 2024.
5. Canadian Institute for Health Information. Children and Youth With Medical Complexity in Canada Methodology Notes. https://www.cihi.ca/sites/default/files/document/children-youth-with-medical-complexity-meth-notes-en.pdf. Published 2020. Accessed June 16, 2025.
6. Canada S. Postal Code Conversion File Plus (PCCF+_ version 8A, Reference Guide. https://library.carleton.ca/sites/default/files/2023-03/PCCF%2BUserguide-2021.pdf. Published December 2022 Postal codes. Accessed June 16, 2025.
7. Roberts SB, Colacci M, Razak F, Verma AA. An Update to the Kaiser Permanente Inpatient Risk Adjustment Methodology Accurately Predicts In-Hospital Mortality: a Retrospective Cohort Study. *J Gen Intern Med.* 2023;38(15):3303-3312.
