# Supplementary material for: Characteristics and Outcomes of Adults Hospitalized With Childhood-Onset Complex Chronic Conditions
Source: JAMA Netw Open. 2026 Jan 28;9(1):e2553610. doi: 10.1001/jamanetworkopen.2025.53610 (PMC12853208; doi:10.1001/jamanetworkopen.2025.53610)
Supplement: Supplement 3. — Data Sharing Statement [file jamanetwopen-e2553610-s003.pdf]

Data Sharing Statement

Malecki. Characteristics and Outcomes of Adults Hospitalized With Childhood-Onset Complex Chronic Conditions. *JAMA Netw Open*. Published January 23, 2026.
doi:10.1001/jamanetworkopen.2025.53610

Data
Data available: No

Additional Information
Explanation for why data not available: Datasets from GEMINI are not permitted to be shared openly given they contain potentially sensitive patient information, based on the data governance policies of the GEMINI research network and its research ethics board-approved study protocols. Data can be accessed in the secure GEMINI research environment. Information about data access can be obtained at www.geminimedicine.ca, by contacting GEMINI.Data@unityhealth.to, or upon request to the corresponding author.
